# Prediction of in-hospital adverse clinical outcomes in patients with pulmonary thromboembolism, machine learning based models

**DOI:** 10.3389/fcvm.2023.1087702

**Published:** 2023-03-14

**Authors:** Yaser Jenab, Kaveh Hosseini, Zahra Esmaeili, Saeed Tofighi, Hamid Ariannejad, Houman Sotoudeh

**Affiliations:** ^1^ Tehran Heart Center, Cardiovascular Diseases Research Institute, Tehran University of Medical Sciences, Tehran, Iran; ^2^ Faculty of Medicine, Tehran University of Medical Sciences, Tehran, Iran; ^3^ Department of Radiology, University of Alabama at Birmingham (UAB), Birmingham, AL, United States

**Keywords:** machine learing, outcome analysis, risk factors, logistic models, gradient boosting machine, pulmonary embolism

## Abstract

**Background:**

Pulmonary thromboembolism (PE) is the third leading cause of cardiovascular events. The conventional modeling methods and severity risk scores lack multiple laboratories, paraclinical and imaging data. Data science and machine learning (ML) based prediction models may help better predict outcomes.

**Materials and methods:**

In this retrospective registry-based design, all consecutive hospitalized patients diagnosed with pulmonary thromboembolism (based on pulmonary CT angiography) from 2011 to 2019 were recruited. ML based algorithms [Gradient Boosting (GB) and Deep Learning (DL)] were applied and compared with logistic regression (LR) to predict hemodynamic instability and/or all-cause mortality.

**Results:**

A total number of 1,017 patients were finally enrolled in the study, including 465 women and 552 men. Overall incidence of study main endpoint was 9.6%, (7.2% in men and 12.4% in women; *p*-value = 0.05). The overall performance of the GB model is better than the other two models (AUC: 0.94 for GB vs. 0.88 and 0.90 for DL and LR models respectively). Based on GB model, lower O_2_ saturation and right ventricle dilation and dysfunction were among the strongest adverse event predictors.

**Conclusion:**

ML-based models have notable prediction ability in PE patients. These algorithms may help physicians to detect high-risk patients earlier and take appropriate preventive measures.

## Introduction

Pulmonary Embolism (PE) causes more than 100,000 cardiovascular-related deaths annually in the United States and is the third leading cause of cardiovascular events after myocardial injury and stroke (1). It occurs approximately 0.5–1 case per 1,000 persons annually, and the incidence rate is dramatically age-dependent, rising sharply after the age of 45 years by the age of 80, the annual average incidence is one case per hundred (2, 3).

Disease outcomes such as in-hospital mortality, major gastrointestinal (GI) bleeding, recurrence, and post-thrombotic syndromes occur differently based on the type of the emboli, which could be massive or sub-massive, and also the underlying comorbidities of patients (4, 5). Studies have shown that the mortality rate in patients with pulmonary embolism without underlying disease is much lower than in patients with cancer, congestive heart failure, or chronic lung disease. However, hemodynamically unstable patients who receive mechanical ventilation or cardiopulmonary resuscitation are at higher risk of mortality (6, 7). Prediction algorithms and risk scoring systems have been developed to help physicians estimate the chance of outcome occurrence in every individual.

The conventional modeling methods are premised on certain assumptions such as linearity and additivity of the data which may not hold true in practice. It is also difficult to model high-dimensional relationships between features with conventional methods. These situations can be addressed using sophisticated machine-learning techniques (8).

In the present study, we aimed to evaluate and compare the performance of two different machine-learning techniques, namely gradient boosting and deep learning with that of the conventional logistic regression method for predicting in-hospital PE adverse outcomes. In addition, selected features with the most association with study main endpoint will be discussed.

## Materials and methods

### Study design and patient selection

In this retrospective analysis conducted in Tehran Heart Center (THC) hospital, Tehran, Iran, all consecutive hospitalized patients diagnosed with pulmonary embolism (based on pulmonary CT angiography) from 2011 to 2019 were recruited. A total number of 1,031 patients were diagnosed at first, 14 patients were excluded due to missing data, and 1,017 patients were finally enrolled in the study. Nearly all patients had at least one post-discharge follow-up. These follow-ups were conducted twice: short and long-term (3 and 12 months following hospital discharge, respectively).

### Study endpoints

The main endpoint of the present study was a composite of the following events: hemodynamic instability and/or all-cause mortality. Hemodynamic instability was defined as low systolic blood pressure that needs inotrope therapy and/or mechanical ventilation in the course of admission.

### Statistical analysis

As it's illustrated in Table 1, continuous and categorical variables were represented as mean and frequencies respectively. Statistical analysis was performed with independent samples *t*-test for continuous numerical variables. Also, a chi-square test was done to evaluate the relationship between categorical variables and final adverse outcomes. The significance level for all of the statistical analyses was determined as a *p*-value of lower than 0.05.

**Table 1:** Intergroup comparison of baseline characteristics.

	Event−(*N *= 919)	EVENT+(*N *= 98)	Odds ratio	95% C.I for odds ratio	*p*-value
**Baseline demographic**					
Sex	Female: 44.3 Male: 55.7	Female: 59.2 Male: 40.8	1.8 (F/M)	1.19–2.78	0.05
Age (years)	58.8	65.4	1.21	1.11–2.17	0.001
Diabetes (%)	18.8	16.3	0.84	0.48–1.4	0.54
Hypertension (%)	40.4	45.9	1.25	0.82–1.9	0.28
Dyslipidemia (%)	24	32.7	1.53	0.9–2.3	0.06
Cigarette smoker (%)	23.2	17.3	0.69	0.4–1.2	0.19
Coronary artery disease (%)	14.7	28.6	2.3	1.4–3.7	0.00
Heart failure (%)	4.5	15.3	3.8	2.05–7.2	0.00
COPD (%)	3.8	5.1	1.35	0.5–3.5	0.53
Drug addiction (%)	4.8	8.2	1.76	0.80–3.8	0.14
Previous CABG (%)	6.5	10.2	1.62	0.8–3.2	0.17
Previous CVA (%)	4.4	7.1	1.69	0.73–3.88	0.21
Malignancy (%)	4.7	8.2	1.81	0.82–3.97	0.13
Previous DVT (%)	8.8	4.1	0.44	0.15–1.2	0.10
Previous PE (%)	6.3	5.1	0.79	0.31–2.0	0.63
Recent immobility (%)	27.4	30.6	1.16	0.74–1.83	0.50
Obesity (BMI > 30) (%)	34.3	25.5	0.65	0.4–1.05	0.08
IV drug user (%)	1	3.1	3.19	1.85–11.9	0.05
Recent surgery (%)	12.7	12.2	0.95	0.59–1.80	0.89
**Signs and symptoms**					
Dyspnea (%)	89	88.8	0.96	0.49–1.8	0.92
Cough (%)	11	11.2	1.02	0.5–1.9	0.94
Hemoptysis (%)	3.9	5.1	1.3	0.5–3.4	0.57
Syncope (%)	10.4	14.3	1.4	0.7–2.6	0.24
Clinical signs of DVT (%)	24.6	18.4	0.7	0.4–1.1	0.17
Altered mental status (%)	3	11.2	4.0	1.9–8.3	0.00
Palpitation (%)	32.9	32.7	0.99	0.6–1.5	0.96
**Physical examination**					
Systolic blood pressure (mmHg)	129.8	124.6	0.95	0.85–0.98	0.02
Heart rate (beat/min)	99.9	107.2	1.18	1.1–3.44	0.001
Respiratory rate (/min)	22.3	24.9	1.31	1.13–2.45	0.001
O_2_ saturation (%)	92.4	86.6	0.91	0.78–0.95	0.001
**Drug history**					
Aspirin (%)	16.9	14.3	0.82	0.45–1.48	0.51
Clopidogrel (%)	2.2	3.1	1.4	0.41–4.8	0.57
Beta blockers (%)	23.4	38.8	2.07	1.34–3.20	0.001
Warfarin (%)	3	3.1	1.005	0.3–3.36	0.99
Statins (%)	37	46.9	1.5	0.99–2.29	0.054
ACEi/ARB (%)	9.7	6.1	0.6	0.25–1.42	0.24
**Laboratory tests**					
Hemoglobin (mg/dl)	13.90	13.3	0.91	0.87–0.93	0.03
White blood cells (/dl)	10,603	12,105	1.24	1.15–1.43	0.001
Platelets (/dl)	226,719	233,369	1.03	0.83–1.34	0.49
Creatinine (mg/dl)	0.93	1.06	1.27	1.15–2.43	0.001
Hs-troponin T (Ng)	59.0	72.1	1.22	0.89–2.32	0.37
**Electrocardiography**					
RBBB (%)	16.9	17.3	1.03	0.59–1.79	0.90
S1S2S3 (%)	14.4	15.3	1.07	0.60–1.92	0.80
Q in V1 (%)	11.2	14.3	1.32	0.72–2.41	0.36
AF rhythm (%)	5.2	17.3	3.80	2.09–6.92	0.00
S1Q3T3 (%)	36.7	35.8	0.95	0.61–1.47	0.83
Right axis deviation (%)	10.5	15.3	1.62	0.90–2.93	0.10
ST-Elevation in V1 (%)	13.8	12.2	0.87	0.46–1.63	0.66
**Pulmonary CT angiography**					
Segmental A (%)	15.1	6.1	0.36	0.15–0.85	0.01
Lobar A (%)	79.2	72.5	0.82	0.45–0.92	0.05
Saddle emboli (%)	12	13.3	1.12	0.60–2.08	0.70
Pleural effusion (%)	13.7	25.5	2.15	1.31–3.52	0.002
RV strain (%)	16.3	28.1	1.50	1.28–1.87	0.01
Pulmonary infarction (%)	15.6	17.3	1.13	0.65–1.97	0.64
**Echocardiography**					
RV/RA Thrombus (%)	3.5	9.2	2.80	1.29–6.06	0.006
PA Thrombus (%)	1	4.1	4.30	1.30–14.23	0.009
PFO (%)	0.5	3.1	5.77	1.35–24.5	0.007
RV Dysfunction (%)	61.9	83.7	3.15	1.81–5.47	0.00
RV Dilation (%)	60	81.6	2.96	1.75–5.03	0.00
LV ejection fraction (%)	51.5	49.9	0.95	0.89–0.98	0.05
**Treatment**					
Contraindication To Fl (%)	3.4	11.2	3.62	1.75–7.45	0.00
Fibrinolysis (%)	14.5	29.6	2.48	1.55–3.97	0.00
AC: LMWH (%)	16.1	4.1	0.22	0.08–0.61	0.002
AC: UFH (%)	83.1	93.9	3.11	1.33–7.23	0.006
AC: NOACs (%)	7.3	1	0.13	0.01–0.95	0.01
Thrombectomy (%)	0.5	9.2	18.48	6.06–56.35	0.00
IVC Filter (%)	2.4	11.2	5.15	2.41–10.98	0.00
Aspirin (%)	43.1	55.1	1.6	1.06–2.46	0.02
Statins (%)	32.6	19.4	0.49	0.29–0.83	0.007
**Complications**					
ICH (%)	0.3	1.0	3.14	0.32–30.5	0.29
GI bleeding (%)	0.9	5.1	6.12	1.96–19.09	0.00
HIT (%)	2.1	4.1	3.19	0.85–11.99	0.07
Blood transfusion (%)	1.7	14.3	9.40	4.43–19.93	0.00
Missed PE (Initial) (%)	7.8	12.2	1.64	0.85–3.14	0.13

COPD, chronic obstructive pulmonary disease; CABG, coronary artery bypass grafting; CVA, cerebrovascular accident; DVT, deep vein thrombosis; PE, pulmonary embolism; BMI, body mass index; ACEi, angiotensin-converting enzyme inhibitors; ARB, angiotensin ii receptor blockers; RBBB, right bundle branch block; RV, right ventricle; LV, left ventricle; PA, pulmonary artery; RA, right atrium; PFO, patent foramen oval; FL, fibrinolytic; AC, anti-coagulant; IVC, inferior vena cava; UFH, unfractionated heparin; LMWH, low molecular weight heparin; NOAC, novel oral anti-coagulant; HIT, heparin-induced thrombocytopenia.

### Data extraction and processing

Demographics and clinical and paraclinical variables were extracted from Electronic Health Records (EHR). In view of the absence of a registry system for PE patients over the study period (2011–2019), the EHR data were manually extracted. A total number of 120 variables were identified. Based on previous similar studies (9–15) and our primary analysis, finally 76 variables remained, and further analysis was done using these selected variables. We divided all the variables into ten categories, including “baseline demographic and past medical history”, “signs and symptoms”, “physical examination”, “drug history”, “laboratory tests”, “electrocardiography findings”, “pulmonary CT angiography findings”, “echocardiography findings”, “treatment options” and finally “complication” variables.

### Missing data

We had missing data for some cases in different variables. Our approach to missing data was a combination of these two methods: (1) Imputation method, or (2) Removal of the data. Our main strategy was data imputation using the K-nearest neighbor (KNN) algorithm if the variable had notable importance for the prediction process and its missing values were few. This is a standard strategy for managing missing data that effectively imputes the expected values instead of the missing ones while having less of an impact on the final analysis than other traditional approaches. On the other hand, the variable could be eliminated if the number of missing values was remarkable and the variable wasn't significant enough (based on primary data analysis for the importance of variables). Furthermore, if a case had a high amount of missing data, we totally removed that case as well (14 cases were totally removed out of 1,031).

### Feature selection

As various scores for the prediction and categorization of PE patients (like PESI, sPESI, Bova, etc.) use different variables from different categories, we tried to include all of them together and evaluate them in combination for one well-structured dataset. This way allowed us to find the most important factors from approximately all diagnostic tools to preciously predict future adverse events for hospitalized PE patients. Of course, since all of these data are prepared within the first few hours of a PE patient's admission to the emergency room, we primarily used the baseline variables, including demographic, physical examination, electrocardiography, echocardiography, laboratory, and imaging data; and our models mostly uses only these data for prediction process. Yet, we decided to include some of the most important complications that might occur during hospitalization, alongside previously mentioned baseline features, because these could potentially change the course of the disease and worsen the patient's prognosis.

The next step was to choose the best variables for model development (after data preprocessing and missing value management). In this step, which is called “Feature Selection”, we used two methods: first, regarding traditional statistical analysis, we determined the variables which had significant differences between the two groups; second, we utilized the more precise method, L1 regularization (or Lasso regression) method, which is one of the best methods for feature selection in data science. L1 regularization lets us find the most important variables for the prediction of final results. Using this method and the traditional analysis, we eventually determined 35 variables that were more important for model development.

### Model development

Eventually, 41 variables of the initial 76 were excluded from future model development. The 35 retained variables for the development of our models included: age, sex, systolic blood pressure, heart rate, respiratory rate, coronary artery disease, heart failure, obesity, intravenous (IV) drug user, altered mental status, O_2_ saturation, hemoglobin, white blood cells, creatinine, CT angiography variables [segmental and lobar artery thrombosis, pleural effusion, right ventricle (RV) strain], echocardiography features [patent foramen oval (PFO), RV dysfunction, RV dilation, pulmonary artery (PA) and RV/RA thrombosis, left ventricular (LV) ejection fraction, treatment variables (contraindication to fibrinolytic, treatment with fibrinolysis/thrombectomy/inferior vena cava filter, anticoagulation with unfractionated heparin (UFH), low molecular weight heparin (LMWH), novel oral anticoagulants (NOACs)] and complication variables [intracranial hematoma (ICH), heparin-induced thrombocytopenia (HIT), GI bleeding, blood transfusion].

The features that the model applies to them are based on the routine index units that are regularly employed by healthcare facilities. These index units for each element are detailed in Table 1. We offer the variables to the model with these typical units, and then it would convert them into interpretable numbers for computing the results automatically on its own.

Then, for the creation of the prediction models, the whole dataset with 1,017 patients was randomly divided into three categories: (1) training set (56% of total population, *n* = 570), (2) validation set (14% of total population, *n* = 141) and (3) testing set (30% of total population, *n* = 306). As a final step, three prediction models were developed using the R programming language: a Gradient Boosting model, a Deep Learning model, and a Logistic Regression model. By learning them through the training set, tuning their hyperparameters with the validation set, and finally fitting the models onto a testing set, performance metrics were determined for each model. The results were then compared to find the most accurate model (Supplementary File).

The Logistic Regression (LR) model is a common method that uses the independent variables (the “predictors”) to predict the class of a categorical type variable (the “target variable”). LR models the probability of one target out of the two possible probabilities (binary LR), using the log-odds (the logarithm of the odds), and the class of the target variable will be predicted based on this probability.

The Gradient Boosting (GB) model is a machine learning method that commonly is used for classification problems. Gradient boosting belongs to the class of ensemble methods, which means it uses multiple models (the “weak models”) and combines them to improve their results and get the best final model. Typically, the weak models are based on a decision tree method. Recently, the GB model has become more popular in medical data analysis due to its high prediction accuracy compared to other machine learning or traditional statistical analyses.

The Deep Learning (DL) model is a sort of neural network which consists of neural layers, and each layer includes some nodes. The nodes in consecutive layers are connected with the “weights” that are set during the learning method and are tuned in the validation process. The output from all of the serial layers is the probability of the target variable, which would be converted to the predicted class regarding previous learnings. Although the deep learning method is usually used for developing prediction models on large datasets, however, it could be applied to datasets of any size, but some techniques should be implemented in such situations to augment the training dataset and improve the final estimations. Our deep learning model in this study was based on a multi-layer perception (MLP) method since the MLP neural networks work well on tabular data. The model consists of 4 layers, including the input layer, two hidden layers (each containing ten nodes), and the output layer.

**Figure 1:**
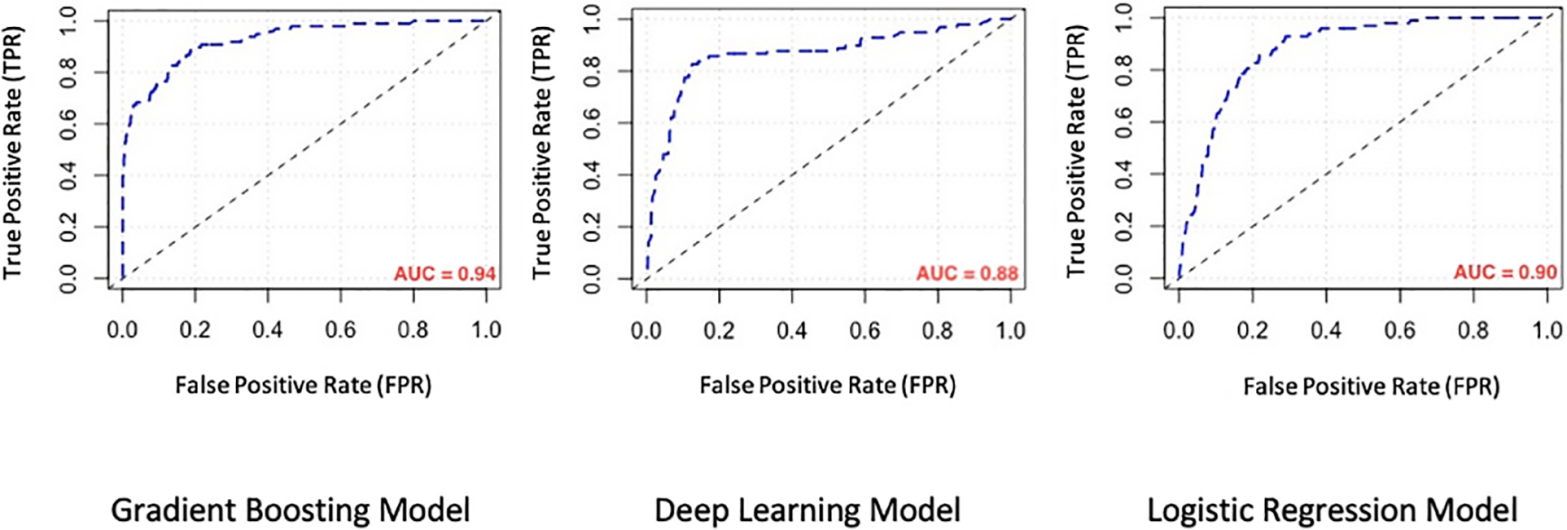
Comparison of the receiver operating characteristic curve (ROC curve) of machine learning models. AUC, area under the curve.

**Figure 2:**
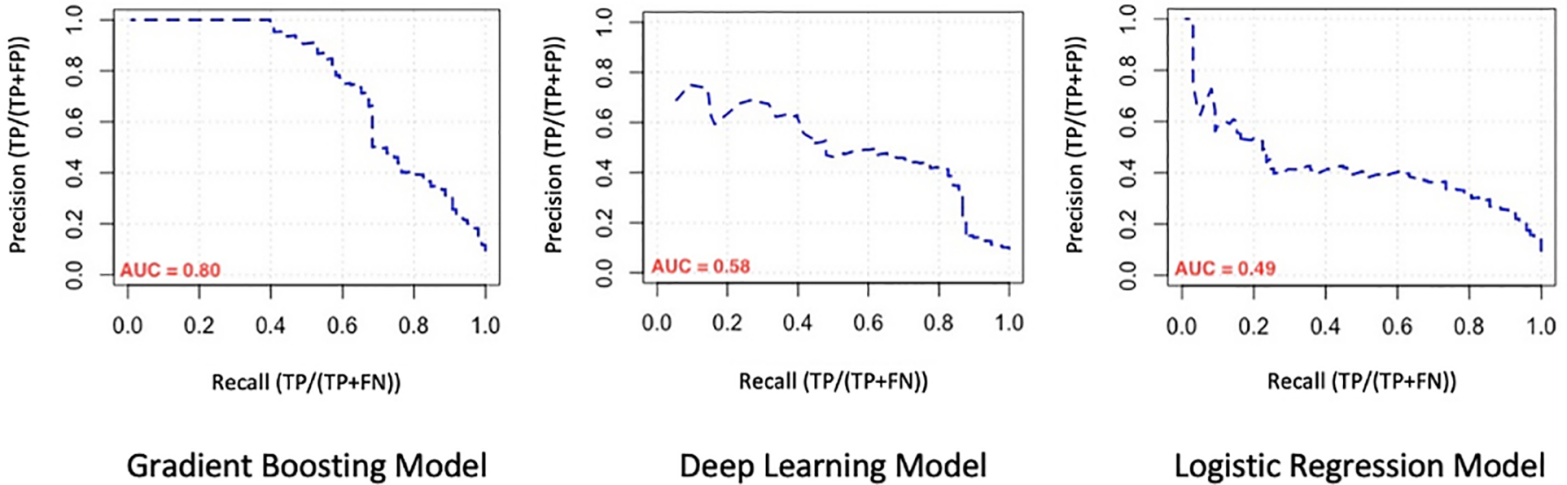
Comparison of the precision-recall curve (PR curve) of machine learning models. AUC, area under the curve.

We evaluated the predictive power of the models on a single testing dataset. For the test set to be able to accurately assess the performance of developed models, it must have the same distribution of adverse outcomes as the whole dataset, so that it mimics the pattern of distribution of events in the main population (i.e., the overall prevalence of final events should be about 9–10 percent). Many factors should be considered for this purpose (called “performance metrics”), such as the Receiver Operating Characteristic curve (ROC curve), the precision-recall curve (PR curve), area under the curve (AUC) of the ROC curve, AUC of the PR curve, accuracy, precision, recall, F1-score and the Matthews Correlation Coefficient (MCC) of the model. The ROC curves and the PR curves of the 3 models are shown in Figures 1, 2 respectively.

Since the target variable (the composite outcome) had two categories, in which the incidence of the events is so fewer than the number of event-free patients, we had a class imbalance problem. To strengthen the estimations, we should convert our training set to a dataset that consists of a more balanced outcome variable (a process that can be done using the data augmentation methods), but the important point is that in such analysis, the validation set and also the testing set should have the same ratio of target variable as the original dataset does have. So first we divided our original data into random samples and created the training, validation, and testing set with respect to preserving the ratio of events in the validation and testing set, then, the data augmentation process for the training set was done using the ROSE (Random Over Sampling Examples) library in R Studio.

## Results

### Basic characteristics

A total number of 1,017 patients were finally enrolled in the study, including 465 women and 552 men. The most common traditional cardiovascular risk factor among patients was hypertension (40.9%), and 14.6% of patients had a history of VTE (including 8.4% for previous DVT and 6.2% for previous PE). The most frequent symptom was dyspnea (89%).

### Main outcome

The overall incidence of study-defined events (composite of adverse outcomes) was 9.6%, (7.2% in men and 12.4% in women) (*p*-value = 0.05). Event-free patients were younger (58.8 ± 17.3 vs. 65.4 ± 15.7 years old), had higher in-admission systolic blood pressure (129.8 vs. 124.6 mmHg), and higher O_2_ saturation (92.4% vs. 86.6%) (*p*-value < 0.05).

### ECG findings

S1Q3T3 pattern (i.e., marked S wave in lead I, inverted T, and marked Q wave in lead III) was the most frequent dynamic change, which was not significantly different between event-positive and event-negative groups (*p*-value 0.83). Atrial fibrillation (AF) rhythm occurred significantly higher in the patients with in-hospital events (17.3% vs. 5.2%, *p*-value = 0.00).

### Echocardiographic findings

In echocardiography examination, 64% of the patients had at least mild right ventricular dysfunction. The mean ejection fraction was slightly (but statistically significant) lower in event-positive patients (49.9 ± 8.7 vs. 51.5 ± 7.4, *p*-value = 0.05). In addition, patients with in-hospital events were more likely to develop RV dysfunction and visible thrombus in the right atrium/right ventricle and pulmonary artery (*p*-value < 0.05).

### Pulmonary CT angiography

Lobar artery involvement was the prevailing pattern of thrombo-emboli in the pulmonary CT angiography of both two groups, however the prevalence of segmental PE was significantly higher in the event-negative group, whereas, the saddle emboli was more prevalent (although non-significant) among the event-positive subjects. Also, evidences of the RV straining in CT angiography sequences were seen more in the event-positive patients (28.1% vs. 16.3%, *p*-value = 0.010).

### Treatment

As we expected, the rate of subjects that had a contraindication for fibrinolytic administration, was significantly higher among the event-positive group (11.2% vs. 3.4%, *p*-value < 0.001). Also, the overall rate of individuals who received fibrinolytic agents was higher in the event-positive group as well (14.5% vs. 29.6%, *p*-value < 0.001). Furthermore, the thrombectomy procedure was performed in 9.2% of patients in the event-positive group, compared with 0.5% of subjects in the event-negative group (*p*-value < 0.001).

### Complications

Among all of the major complications, the incidence of gastrointestinal bleeding and also the need for blood transfusion were significantly higher in the event-positive group compared to the event-negative group (5.1% vs. 5.1%, *p*-value < 0.001 and 14.3% vs. 1.7%, *p*-value < 0.001, respectively).

### Evaluation of model performance

In order to compare model performances to predict mentioned adverse outcomes in our population, performance metrics associated with model evaluation are demonstrated in Table 2. As seen there, the overall performance of the GB model is better than the other two models (AUC: 0.95 for GB vs. 0.91 and 0.92 for DL and LR models respectively). Because the distribution of composite outcome in the population was so imbalanced, interpretation of the model only based on the AUC of the ROC-curve is not enough, so we considered comparing the AUC of PR-curve models because it reflects the performance of models in an imbalanced population more accurate. On the other hand, while the “accuracy” factor is not appropriate for imbalanced datasets, the Matthews Correlation Coefficient (MCC) is more interpretable and accurate for evaluating the prediction models in such populations. As we expected from previous results, the MCC of the GB model was at a higher level in comparison with the other two models. So based on what we observed, the overall performance of the GB model for determination and prediction of the composite adverse outcomes group is higher than the other DL and LR models.

**Table 2:** Performance metrics for comparison of the three models.

Model	AUC-ROC	AUC-PR	Accuracy	MCC	F1-score
**Logistic regression**	0.90	0.49	0.94	0.55	0.58
**Deep learning**	0.88	0.58	0.93	0.62	0.64
**Gradient boost**	0.94	0.80	0.96	0.74	0.76

AUC, area under the curve; ROC, receiver operating characteristics; PR, precision-recall; MCC, Matthews correlation coefficient.

### SHAP analysis

**Figure 3:**
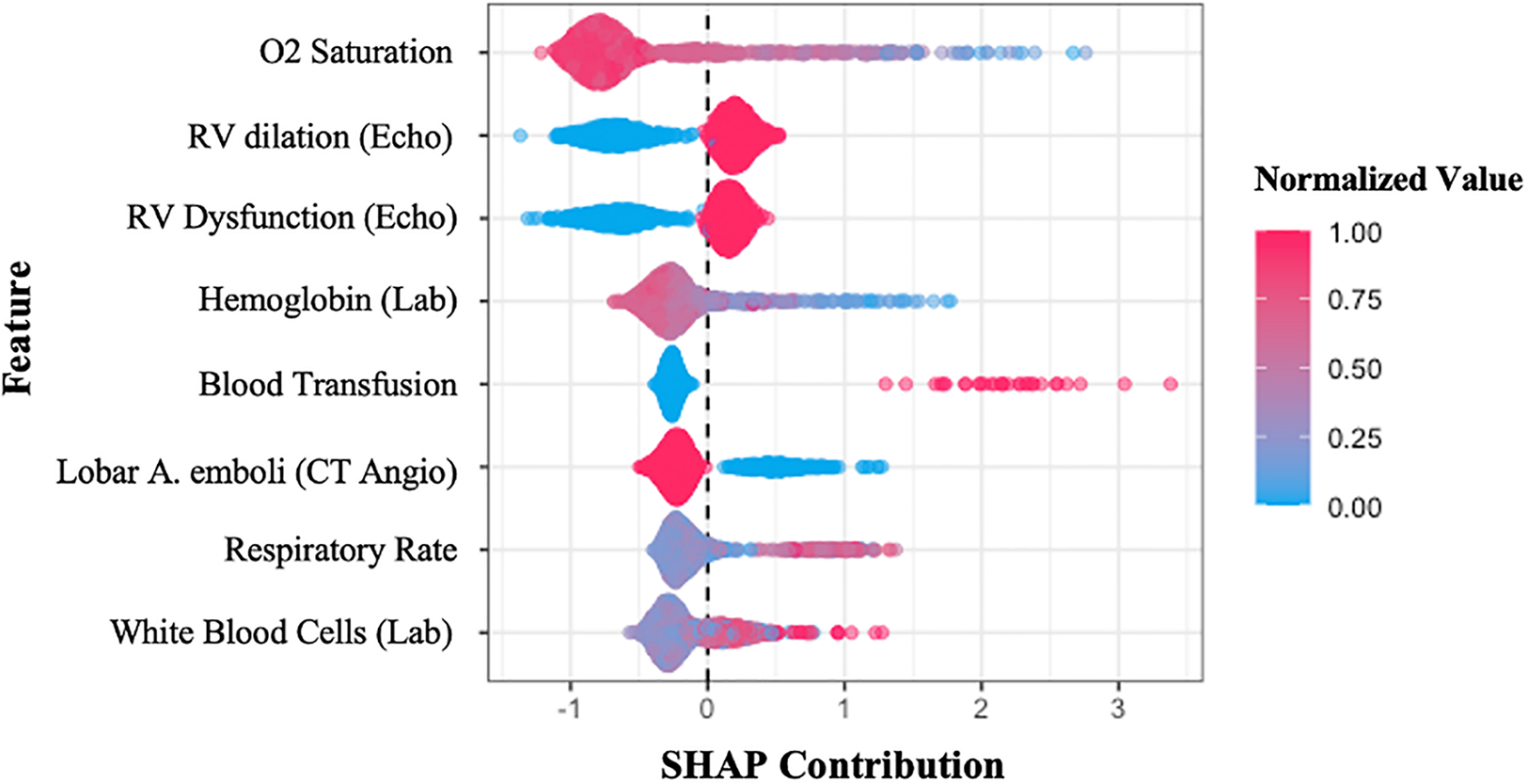
*SHAP summary plot* sorts the features used in the gradient boosting model, concerning their importance in the final decision-making process. The *y*-axis shows the variables, while the *x*-axis represents the Shapley value of each variable. The colors illustrate the relationship between features and prediction (composite adverse outcomes), red shows the direct relationship, whereas blue means inverse relation. SHAP, SHapley Additive exPlanations; Echo, echocardiography; Lab, laboratory test; CT angio, pulmonary CT angiography.

In order to identify what features mostly impact the final prediction, the “SHAP” analysis was done on our GB model. This analysis sorts the features based on their importance for algorithm decision-making. Furthermore, it demonstrates the variable's “Shapley Value”, a value that determines the contribution of a player in the game theory, whereas, in machine learning, it reflects how the system makes the final decision using each of the variables. In our survey, as shown in Figure 3, the most important variables for the GB model to predict the occurrence of adverse events, in order of their importance, are lower O2 saturation (the most important), RV dilation, and RV dysfunction in echocardiography images (which their existence is contributed to a greater risk for events), lower levels of hemoglobin, higher need for blood transfusion, absent of only the lobar artery emboli (which means that the patient had a saddle emboli and as we expected, is at a higher risk for adverse outcomes), higher respiratory rate, and higher levels of leukocytosis in laboratory tests.

## Discussion

ML is commonly used to develop predictive models for medical datasets and their performance generally surpasses that of conventional methods when dealing with high-dimensional relationships between features.

We developed 3 machine learning models and compared their performance for predicting in-hospital PE adverse outcomes, namely the Gradient boosting model, the Deep learning model, and the Logistic regression model. Of the three methods, gradient boosting achieved the highest AUC (higher than LR).

**Figure 4:**
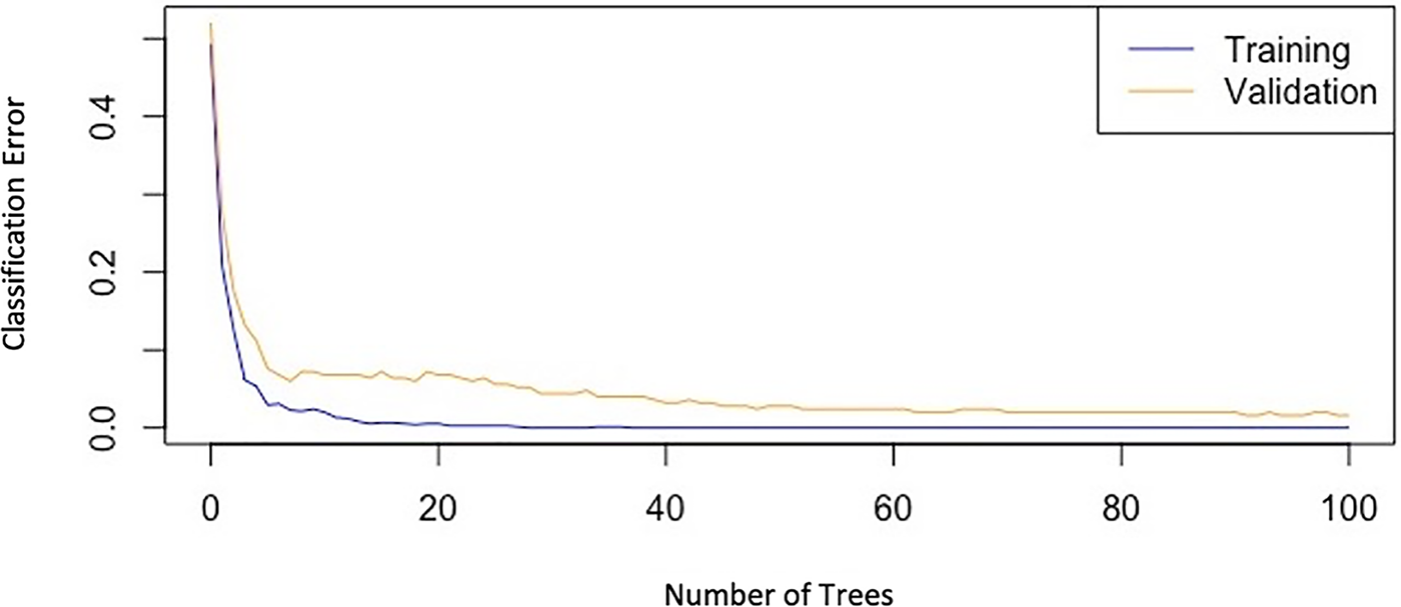
Graph of classification-error of the gradient boosting model versus the number of trees, showing the decreasing trend of errors in both training and validation datasets.

Among the many scores that have been proposed for PE outcomes, the pulmonary embolism severity index (PESI) is the most widely used one. However, many important predictors such as echocardiographic parameters and lab data have not been included in PESI which may limit its ability in subgroups of patients. This is where ML-based models which are based on the pile of electronic medical records and artificial intelligence come to work. It has been demonstrated in several studies (16–18) that PESI score's overall predictive power is modest, with the ROC plot AUC ranges from 0.66 to 0.77, whereas our best model's AUC was 0.94; Of course, it needs to be tested on other external datasets as well to confirm. So, we decided not to do this comparison because it seemed to be imprecise due to the lack of external validation for our AUC. Instead, we compared the performances of our various models on the data (Figure 4). It is noteworthy to mention that our model mostly utilizes baseline parameters for the prediction process, which are available in the emergency room during the initial hours of patient admission, nonetheless, it also includes a few non-baseline variables (including ICH, HIT, GI bleeding, and blood transfusion) which may arise in the ensuing days of hospitalization.

Eight features were included in our final models that are mostly different from what we calculate in the PESI score. Detailed discussion about these features will come afterward.

1. O_2_ saturation was the leading feature identified. Our analysis shows a direct relationship between decreased O_2_ saturation and adverse outcomes in PE, which is compatible with previous studies. As demonstrated in an earlier study, oxygen saturation <88% is associated with the severity of PE (19).
2. RV dilation and dysfunction were also identified as important variables for predicting adverse outcomes using the GB model. Recent studies show that RV dilation assessed using an RV/LV ratio greater than 0.9 leads to a greater risk for mortality in PE patients (20). Another analysis has assessed RV failure in PE patients using echocardiography and spiral CT or by increased levels of BNP, pro-BNP or troponin-T and also showed that it is a significant risk factor for mortality in PE patients which is in keeping with our findings (21).
3. Considering anemia as an independent risk factor for developing PE is challenging. Some studies report a positive correlation between anemia and PE due to decreased blood viscosity resulting in reduced secretion of anti-thrombotic mediators (22), while others found the opposite (23). In terms of analyzing the low hemoglobin (Hb) level effect on PE outcomes, our analysis was in correlation with past studies and showed that low Hb level could predict adverse outcomes in PE patients. A study reported a strong association between anemia and PE mortality and reports a higher risk in patients who have RV dysfunction along with anemia (24).
4. Our analysis showed that blood transfusion in patients was related to a higher risk of developing adverse outcomes. Transfusions are often associated with anemia, an independent risk factor for mortality in patients. On the other hand, blood transfusion increases the risk of infection and develops a hypercoagulable state which can worsen the situation. Similar to our findings, one study has assessed packed-cell transfusion in PE patients and defined it as a significant independent predictor for short-term and long-term mortality (25). However, this is one of the few non-baseline variables the model uses for the prediction task, which might occur during the hospitalization period and essentially couldn't be assigned at the first few hours of patient admission.
5. Saddle embolus was reported higher in our outcome group compared to our event-free group, but this was not statistically significant, probably because of the lower number of saddle emboli than our lobar emboli and segmental emboli patients. Recent studies show that saddle pulmonary embolism results in a higher proportion of hemodynamic instability and other adverse events (26, 27). Despite saddle emboli, lobar artery emboli patients had a higher proportion in our population in both groups, so it worked as a helpful variable for outcome prediction. Patients with proximal PE are at a higher risk than those with subsegmental PE. It is caused by pulmonary hypertension and enlargement of the right ventricle with more proximal localization of emboli (28). Similar findings were obtained in previous research (29, 30). A meta-analysis assessed the prognostic role of emboli burden in PE patients using CTA and reported an increased risk of 30-day mortality in patients with an embolism in the central branches of pulmonary arteries (31).
6. Patients with adverse events tend to have higher respiratory rates compared to the event-free group. A similar study tried to build a predictive model for PE and introduced both low O_2_ saturation and respiratory rate ≥30 as significant predictors for mortality and other adverse effects in PE (32).
7. Leukocytosis is associated with poorer outcomes in many cardiovascular diseases such as acute coronary syndrome, ischemic stroke, and pulmonary embolism (33, 34). White blood cell (WBC) count is considered as a parameter for adverse outcome prediction in our model, which is in line with a retrospective cohort study that assessed the prognostic impact of WBC count in PE patients (35–37). An explanation for this association is that leukocytosis in PE usually happens when there is an infiltration around a myocyte injury in RV which is an indicator for PE-related RV dysfunction, a known predictor for adverse outcomes (38).

This study had some limitations. First, single-center studies always are threatened by a lack of generalizability. However, Tehran Heart Center is a referral hospital in Iran, and many patients from diverse socio-demographic features are admitted. Second, the sample size was not high enough to precisely test and train the model. The power of the study was not high due to the low event rate. Further research with a larger sample size is needed in this field. On the other hand, the absence of external validation is another limitation of this study. Our clinic is one of the few national referral centers for PE patients, and other centers don't have data as organized as our center does for these patients. As a result, we were unable to obtain other structured data for accurate external validation. Nevertheless, we are discussing with centers in other countries over this issue. Ultimately, the use of non-baseline parameters in the models limits their utility at the very first hours of initial medical contact. To further our work, we intend to eliminate these variables while still sustaining a high level of model performance.

## Conclusion and future prospective

ML-based models have notable prediction ability in PE patients. Personalized medicine is the main area that may benefit from these algorithms. These algorithms may help physicians to detect high-risk patients earlier and take appropriate preventive measures.

## Data Availability

The data analyzed in this study is subject to the following licenses/restrictions: The data that support the findings of this study are available on request from the corresponding author (ST). The data are not publicly available due to containing information that could compromise research participant privacy/consent. Requests to access these datasets should be directed to ST, saeedtofighi69@gmail.com.
