# Supplementary material for: Prediction of in-hospital adverse clinical outcomes in patients with pulmonary thromboembolism, machine learning based models
Source: Front Cardiovasc Med. 2023 Mar 14;10:1087702. doi: 10.3389/fcvm.2023.1087702 (PMC10043172; doi:10.3389/fcvm.2023.1087702)
Supplement: Supplementary file 1 [file Table1.docx]

**The machine-learning models and H2O package in R**

Here we present our models’ codes with a brief explanation for each part. The codes are based on the R programming language, which can be run in RStudio (version 2022.02.3) environment. As it’s obvious in these codes, we used the “H2O” package for the development of our models. The H2O package is an open-source machine-learning platform that provides many supervised and unsupervised algorithms including regression models, gradient boosting machines, neural network and deep learning, ensemble models, PCA, K-means, etc. It also lets us implement our model using the H2O Auto-ML module, which is a fully automatic machine-learning algorithm. Furthermore, we used the grid search method in this library, which trains and validates the models created with different combinations of hyperparameters and provides us with a list of models with their performance results. Then we can choose the best one regarding the highest performance metrics. Eventually, we test the selected model with the testing set and get the performance metrics based on its confusion matrix.

**The Codes**

***### Loading Libraries and the Dataset file***

library(ROSE)

library(dplyr)

library(h2o)

df = read.csv("/Users/Desktop/Data.csv")

***### Min-Max scaler***

min_max <- function(x)

{

return((x- min(x)) /(max(x)-min(x)))

}

df$Demog.Age = min_max(df$Demog.Age)

df$ClinicFind.SBP = min_max(df$ClinicFind.SBP)

df$ClinicFind.HR = min_max(df$ClinicFind.HR)

df$ClinicFind.RR = min_max(df$ClinicFind.RR)

df$Symptoms.O2.sat = min_max(df$Symptoms.O2.sat)

df$X1stLab.Hb = min_max(df$X1stLab.Hb)

df$X1stLab.Cr = min_max(df$X1stLab.Cr)

df$X1stLab.Plt = min_max(df$X1stLab.Plt)

df$X1stLab.WBC = min_max(df$X1stLab.WBC)

df$Lab.hsTnT = min_max(df$Lab.hsTnT)

df$Echo.LVEF = min_max(df$Echo.LVEF)

***### Train-Test Splitting***

sam = sample(x = nrow(df), size = (nrow(df)*0.70))

train = df[sam,]

test = df[-sam,]

***### H2O initiation and conversion of datasets***

h2o.init()

x = c(1:35)

y = 36

train = as.h2o(train)

test = as.h2o(test)

***### Train-Valid Splitting***

splits <- h2o.splitFrame(data = train, ratios = c(0.80), seed = 1)

train <- splits[[1]]

valid <- splits[[2]]

***### Data augmentation for the class imbalance problem***

train = ROSE(Composite.Outcome~. , data = train, N = 1000)$data

***### Gradient Boosting Model***

gbm_params <- list(learn_rate = c(0.01, 0.1),

max_depth = c(3, 5, 9),

sample_rate = c(0.8, 1.0),

col_sample_rate = c(0.2, 0.5, 1.0))

gbm_grid <- h2o.grid("gbm", x = x, y = y,

grid_id = "gbm_grid",

training_frame = train,

validation_frame = valid,

ntrees = 100,

seed = 1,

hyper_params = gbm_params)

gbm_gridperf <- h2o.getGrid(grid_id = "gbm_grid",

sort_by = "auc",

decreasing = TRUE)

best_gbm <- h2o.getModel(gbm_gridperf@model_ids[[1]])

best_gbm_perf <- h2o.performance(model = best_gbm, newdata = test)

best_gbm_perf

***### Neural Network Model***

activation_opt <- c("Rectifier", "Maxout", "Tanh")

l1_opt <- c(0, 0.00001, 0.0001, 0.001, 0.01)

l2_opt <- c(0, 0.00001, 0.0001, 0.001, 0.01)

hyper_params <- list(activation = activation_opt, l1 = l1_opt, l2 = l2_opt)

search_criteria <- list(strategy = "RandomDiscrete", max_runtime_secs = 600)

dl_grid <- h2o.grid("deeplearning", x = x, y = y,

grid_id = "dl_grid",

training_frame = train,

validation_frame = valid,

seed = 1,

hidden = c(10,10),

hyper_params = hyper_params,

search_criteria = search_criteria)

dl_gridperf <- h2o.getGrid(grid_id = "dl_grid",

sort_by = "AUC",

decreasing = TRUE)

best_dl_model_id <- dl_gridperf@model_ids[[1]]

best_dl <- h2o.getModel(best_dl_model_id)

best_dl_perf <- h2o.performance(model = best_dl, newdata = test)

best_dl_perf

***### Logistic Regression Model***

glm_params <- list(alpha= c(0.0, 0.2, 0.4, 0.6, 0.8, 1.0))

glm_grid <- h2o.grid("glm", x = x, y = y,

grid_id = "glm_grid",

training_frame = train,

validation_frame = valid,

seed = 1,

hyper_params = glm_params)

glm_gridperf <- h2o.getGrid(grid_id = "glm_grid",

sort_by = "logloss",

decreasing = TRUE)

best_glm <- h2o.getModel(glm_gridperf@model_ids[[1]])

best_glm_perf <- h2o.performance(model = best_glm, newdata = test)

best_glm_perf

**The confusion matrices**

Tables 1, 2, and 3 illustrate the confusion matrices from the fitting of three machine-learning models on the testing dataset. The total number of patients in the testing set was 306, of which 32 subjects had composite outcomes; so the ratio of events in the testing set is 10.4%, which is approximately the same as the rate of events in the total population (9.6%).

Table 1. Confusion matrix of the Gradient Boosting model

| ***Gradient Boosting model*** | | **Predicted** | |
| --- | --- | --- | --- |
|  |  | No event | event |
| **Actual** | No event | 267 | 7 |
|  | event | 9 | 23 |

Table 2. Confusion matrix of the Deep Learning model

| ***Deep Learning model*** | | **Predicted** | |
| --- | --- | --- | --- |
|  |  | No event | event |
| **Actual** | No event | 253 | 21 |
|  | event | 8 | 24 |

Table 3. Confusion matrix of the Logistic Regression model

| ***Logistic Regression model*** | | **Predicted** | |
| --- | --- | --- | --- |
|  |  | No event | event |
| **Actual** | No event | 245 | 29 |
|  | event | 8 | 24 |

**The Logistic Regression model**

As our main goal for this study was to compare machine learning models with the logistic regression model, and finally our gradient boosting model performance was better than others, we assessed and reported its function with more detail, and did not provide further information about the deep learning and the logistic regression models. However, comprehensive detail about these models is ready to be shared with those who want to know about them. Our logistic regression method’s estimate, standard error, and p-value for each variable are reported in Supplementary Table 4.

*Table 4. The Logistic Regression Model*

| **Variable** | **Estimate** | **SE** | **P-value** |
| --- | --- | --- | --- |
| **Sex (Female)** | 1.13 | 0.59 | 0.055 |
| **Age** | 0.58 | 0.20 | 0.004 |
| **Systolic Blood Pressure** | -0.11 | 0.01 | 0.298 |
| **Heart Rate** | -0.71 | 0.02 | 0.725 |
| **Respiratory Rate** | 0.23 | 0.03 | 0.562 |
| **Past history of CAD** | 0.19 | 0.06 | 0.752 |
| **Past history of HF** | 2.3 | 0.92 | 0.008 |
| **O2 saturation** | -0.89 | 0.03 | 0.004 |
| **Altered mental status** | 0.49 | 0.81 | 0.542 |
| **Obesity** | 0.01 | 0.05 | 0.997 |
| **IVDU** | 4.63 | 2.23 | 0.025 |
| **Hemoglobin** | -1.12 | 0.12 | 0.323 |
| **White blood cells** | 0.22 | 0.06 | 0.001 |
| **Creatinine** | 0.09 | 0.72 | 0.902 |
| **Segmental A. thrombosis** | -4.61 | 1.63 | 0.004 |
| **Lobar A. thrombosis** | -2.28 | 0.80 | 0.004 |
| **Pleural Effusion** | 1.59 | 0.69 | 0.021 |
| **RV strain** | 2.12 | 0.81 | 0.008 |
| **PFO** | 0.67 | 0.64 | 0.025 |
| **RV Dilation** | 1.78 | 0.69 | 0.052 |
| **RV Dysfunction** | 1.24 | 0.76 | 0.019 |
| **PA thrombosis** | 1.85 | 1.43 | 0.196 |
| **RA/RV thrombosis** | 1.10 | 1.18 | 0.307 |
| **LVEF** | 0.55 | 0.36 | 0.132 |
| **Contraindication to fibrinolytic** | 1.22 | 0.77 | 0.114 |
| **Fibrinolysis administration** | 1.09 | 0.62 | 0.080 |
| **Thrombectomy** | 2.57 | 1.51 | 0.089 |
| **IVC filter implantation** | 0.77 | 1.29 | 0.547 |
| **UFH** | -0.98 | 0.87 | 0.289 |
| **LMWH** | -1.35 | 1.09 | 0.216 |
| **NOACs** | 1.45 | 1.66 | 0.383 |
| **ICH** | 5.03 | 2.05 | 0.014 |
| **HIT** | 0.11 | 1.92 | 0.942 |
| **GI bleeding** | 3.18 | 1.51 | 0.035 |
| **Blood transfusion** | 2.91 | 0.98 | 0.003 |
